# Workers’ Compensation Status Confers a Greater Number of Postoperative Visits After Common Upper Extremity Surgeries

**DOI:** 10.7759/cureus.14629

**Published:** 2021-04-22

**Authors:** Tyler W Henry, Clay B Townsend, Pedro K Beredjiklian

**Affiliations:** 1 Orthopaedic Surgery, Sidney Kimmel Medical College, Thomas Jefferson University, Philadelphia, USA; 2 Division of Hand Surgery, Rothman Orthopaedic Institute, Philadelphia, USA

**Keywords:** workers’ compensation, insurance, postoperative, visits, hand surgery

## Abstract

Background

The impact of Workers’ Compensation (WC) status on postoperative healthcare utilization in hand and wrist surgery clinical practice is presently unclear. The purpose of this study was to compare the number of postoperative visits in WC to non-WC patients after common upper extremity surgical procedures.

Methodology

All patients who underwent one of four common surgical procedures (carpal tunnel release, De Quervain’s release, cubital tunnel release, and trigger finger release) between 2016 and 2019 were identified. A total of 64 surgeries billed under WC were randomly selected and matched 1:1 to surgeries billed outside of WC based on the primary CPT code.

Results

The most common procedure was carpal tunnel release (42 patients), followed by trigger finger release (30 patients), cubital tunnel release (28 patients), and De Quervain’s release (16 patients). The average number of postoperative visits was 2.3 (median = 2, range: 1-9) and was significantly higher in the WC group (mean/median = 3.0/3 versus 1.5/1, p < 0.001). Within the 90-day global postoperative billing period, the mean number of visits was 2.2 (median = 2, range: 1-4) in the WC group and 1.4 (median = 1, range: 1-3) in the non-WC group (p < 0.001). The average time to clinical discharge in the WC group was 101 days (range: 10-446 days), and in the non-WC group was 40 days (range: 7-474 days) (p < 0.001). Five patients (7.8%) in the WC group and four patients (6.3%) in the non-WC group were seen for unplanned visits after clinical discharge.

Conclusions

WC status conferred more postoperative visits after common upper extremity surgical procedures, both within and beyond the global billing period. Further investigation and targeted strategies are required to address the observed increase in healthcare utilization.

## Introduction

The impact of Workers’ Compensation (WC) insurance status on the clinical outcomes after surgery has been well described in the literature. After upper extremity surgery, WC status can negatively impact postoperative compliance [1], functional outcomes [2-4], symptom relief [5], return to work [6,7], and patient satisfaction [8,9]. WC status can also place additional strain on the treating orthopedic practice and the broader healthcare system. For example, WC patients in hand and wrist surgical practice have been shown to require a higher number of clinical visits and more diagnostic testing prior to surgery than non-WC patients [10]. The impact of WC status on resource allocation during the postoperative period is less clearly understood.

WC status could represent a substantial resource burden for treating surgeons and affected patients if it confers a higher number of clinical visits after surgery, particularly within the 90-day postoperative global billing period. We therefore sought to compare the number of postoperative visits after common upper extremity surgical procedures between WC and non-WC patients. We hypothesized that WC patients would be seen for a significantly greater number of total postoperative visits, with specific attention to the global billing period. We secondarily assessed the number of unplanned visits, complications, and days to clinical discharge after surgery.

## Materials and methods

Following Institutional Review Board approval, with a waiver of informed consent per institutional protocol, a database query was performed to identify all patients who underwent one of four common upper extremity surgical procedures between 2016 and 2019. The included procedures were trigger finger release (CPT Codes: 26145, 26055), open or endoscopic carpal tunnel release (CPT Codes: 64721 and 29848), cubital tunnel release with or without ulnar nerve transposition (CPT Code: 64718), and De Quervain’s release (CPT Code: 25000). All surgeries were performed by one of 14 fellowship-trained hand surgeons within a single regional orthopedic group in the northeastern United States. The internal billing database was then queried to identify the insurance type billed for all surgical procedures. A total of 352 surgeries billed under WC were identified. Based on a power analysis to identify an effect size of 0.5 at a power of 0.8 and 95% significance level, 64 WC surgeries were selected using a random number generating sequence and matched 1:1 to surgeries billed outside of WC based on the primary CPT code. Matched surgeries were also selected using a random number generating sequence. In all cases, the exact postoperative protocol was subject to the individual treating surgeon’s discretion and any insurance requirements in the WC group.

Electronic medical records were reviewed to collect patient demographics and postoperative courses. All postoperative visits within the treating surgeon’s office were reviewed. This review and subsequent analysis did not include physical therapy sessions. Any subsequent visit related to the operative site after a documented clinical discharge was considered an unplanned visit. Additionally, unscheduled interim visits before clinical discharge for a new or worsening complaint outside of routine follow-up were also considered unplanned visits. The total number of postoperative visits and the number of unplanned visits were recorded for each patient. Any complications or reoperations were also recorded.

After testing for normality using the Shapiro-Wilk test, mean differences were compared using the Mann-Whitney U-test and Kruskal-Wallis test. The rates of unplanned visits and complications were compared using the Chi-square test. Statistical significance was maintained at p < 0.05 for all testing.

## Results

Patient demographics were similar between the two groups, except a lower mean age (54.3 years old versus 64.4 years old, p < 0.001) and a lower percentage of females (43.8% versus 65.6%, p = 0.013) among WC patients (Table 1).

**Table 1:** Demographic characteristics of the study population stratified by WC status. WC: workers’ compensation

Demographic variable	Mean (range)		P-Value
	WC	Non-WC	
Age	54.3 (29–72)	64.4 (24–88)	<0.001
Body mass index	30.7 (21.0–48.1)	31.4 (18.9–49.8)	0.524
	Frequency (%)		
	WC	Non-WC	
Sex			0.013
Female	28 (43.8)	42 (65.6)	
Male	36 (56.3)	22 (34.4)	
Current smoker	7 (10.9)	6 (9.4)	0.616
Diabetes	10 (15.6)	13 (20.3)	0.545
Hypertension	19 (29.7)	21 (32.8)	0.802
Hyperlipidemia	19 (29.7)	23 (35.9)	0.525
Heart disease	4 (6.3)	10 (15.6)	0.152
Rheumatoid arthritis	2 (3.1)	5 (7.8)	0.438
Anxiety	10 (15.6)	9 (14.1)	0.700
Depression	7 (10.9)	9 (14.1)	0.644

Insurance types in the non-WC group included Preferred Provider Organization (27 patients), Medicare (26 patients), Health Maintenance Organization (10 patients), and Federal (one patient). The most common procedure was carpal tunnel release (42 patients) (Table 2).

**Table 2:** Frequency of included procedures.

Procedure	Frequency (%)
Carpal tunnel release	42 (32.8)
Trigger finger release	30 (23.4)
Cubital tunnel release	28 (21.9)
De Quervain’s release	16 (12.5)
Carpal and cubital tunnel releases	6 (4.7)
Carpal and De Quervain’s releases	2 (1.6)
Carpal and trigger finger releases	2 (1.6)
De Quervain’s and trigger finger releases	2 (1.6)

The mean number of total postoperative visits was 2.3 (median = 2, range: 1-9) and was significantly higher in the WC group (mean/median = 3.0/3 versus 1.5/1, p < 0.001). Within the 90-day global postoperative billing period, the mean number of visits was 2.2 (median = 2, range: 1-4) in the WC group and 1.4 (median = 1, range: 1-3) in the non-WC group (p < 0.001). The average time to clinical discharge in the WC group was 101 days (range: 10-446 days), and in the non-WC group was 40 days (range: 7-474 days) (p < 0.001). Five patients (7.8%) in the WC group and four patients (6.3%) in the non-WC group were seen for unplanned visits after clinical discharge (p = 1.00). Reasons for unplanned visits included pain (two WC, one non-WC), wound concerns (one WC, two non-WC), and persistent symptoms (two WC, one non-WC).

The average number of postoperative visits did not differ significantly based on procedure type (p = 0.078) (Table 3). There were no reoperations in either group, and there were a total of eight complications. Superficial infection was the most common complication, occurring in four patients (two WC, two non-WC). The complication rates did not differ significantly between WC and non-WC patients (9.4% versus 3.1%, p = 0.273) (Table 4).

**Table 3:** Mean number of postoperative visits stratified by procedure type. WC: workers’ compensation

Procedure	Mean number of postoperative visits (range)		
	Total	WC	Non-WC
Carpal tunnel release	2.2 (1–7)	3.0 (1–7)	1.5 (1–3)
Trigger finger release	1.6 (1–3)	1.9 (1–3)	1.3 (1–3)
Cubital tunnel release	2.7 (1–8)	3.5 (1–8)	1.9 (1–3)
De Quervain’s release	2.2 (1–6)	3.4 (1–6)	1 (1)
Carpal and cubital tunnel releases	3.5 (1–9)	5.0 (2–9)	2.0 (1–3)
Carpal and De Quervain’s releases	1.5 (1–2)	2	1
Carpal and trigger finger releases	2.5 (2–3)	3	2
De Quervain’s and trigger finger releases	4.0 (2–6)	6	2

**Table 4:** Complication profile stratified by WC status. WC: workers’ compensation

Complication	Frequency	
	WC	Non-WC
Superficial infection	2	2
Wound dehiscence	2	0
Contracture	1	0
Complex regional pain syndrome	1	0

Across both groups, experiencing a complication led to a higher mean number of total postoperative visits compared to those who did not experience a complication (3.0 (range: 2-4) versus 2.2 (range: 1-9), p = 0.018). Within each group, neither age nor other demographic variables were associated with a higher number of postoperative visits (all p > 0.05).

## Discussion

Among common upper extremity surgical procedures, WC status conferred a higher number of total postoperative clinical visits and a higher number of visits within 90 days after surgery. WC patients also remained under clinical care for a significantly longer period of time than non-WC patients. Particularly considering the global billing period during which many of the additional clinical visits occurred, the current findings demonstrate a resource strain for the treating orthopedic practice and a higher resource burden for the WC patients.

The primary finding of this study that WC patients were seen for a higher number of postoperative visits aligns with previously identified trends among hand surgery patients. Day et al. comparatively reported the outcomes of 116 WC patients to nearly 1,300 non-WC patients within a hand and wrist surgical practice. In their study cohort, WC patients required a higher number of clinical visits before surgery once operative intervention was recommended and required more diagnostic testing than non-WC patients [10]. The increased healthcare utilization by WC patients observed in this study, and by Day et al., aligns with the broader literature [11] that has prompted more proactive strategies to monitor and manage resources for these patients [12]. The basis of increased postoperative visits among WC patients is certainly multifactorial, and is influenced by both intrinsic patient factors and insurance requirements. There were undoubtedly multiple instances within our WC cohort in which a clinical visit was stipulated by insurance rather than a clinical necessity or patient preference. Given this challenging complexity, it benefits the treating surgeon to proactively counsel and carefully manage WC patients to limit additional healthcare utilization after surgery.

Though not directly assessed in the present study, functional outcomes and patient satisfaction are also important considerations when attempting to improve the quality and efficacy of care for WC patients. In a systematic review of carpal tunnel release outcomes, Dunn et al. found that WC patients were 16% less likely to return to their preinjury work status [13], and decreased patient satisfaction is an evidenced consequence of WC status [9]. These factors are not only important for appropriate preoperative counseling but also a consideration for potentially prolonged postoperative clinical courses intertwined with poor satisfaction and suboptimal outcomes. The contributory causes towards these associations are again unclear and likely multifactorial. Further explorations into how these potential adverse outcomes can be better managed or prevented are certainly warranted.

Given the higher rate of healthcare utilization by WC patients after upper extremity surgery, and the ever-present need to manage costs among orthopedic practices, strategies must be implemented to better manage resource allocation while improving the experience for these patients. One potential avenue is through the use of telehealth services, which have already increased exponentially after the coronavirus disease 2019 pandemic [14]. Telehealth utilization has been shown to decrease resource burden for clinical practices [15-18], save patients time and money [19-21], and could provide an elegant solution to offset the increased postoperative healthcare utilization demonstrated among WC patients. Telehealth has already shown clinical efficacy in postoperative care after carpal tunnel release [22], and could likely be implemented seamlessly for other common soft-tissue upper extremity surgeries included in the present study. Decreasing the resource burden for WC patients through the convenience of telehealth visits could also potentially improve postoperative satisfaction, which, as stated, is historically poor within this population [9].

This study is not without limitations, most notably its retrospective design which limited the standardization of postoperative protocols between the cohorts. This allowed for potential confounders contributing to the higher rate of visits observed among WC patients, though we estimate the likelihood of this to be small. Specifically, there were demographic differences between the two groups that could have impacted the observed results. Also attributable to this study’s retrospective nature, we were unable to account for patients who may have presented to an outside orthopedic clinic or emergency department during the study period. Additionally, the population size should be considered with the interpretation of our statistical findings for secondary outcomes. We were likely underpowered to detect potential differences between the rates of unplanned visits and complications in WC and non-WC patients. And finally, our findings may not reflect WC patterns of orthopedic practices in other regions.

## Conclusions

In conclusion, WC patients were seen for more postoperative visits after common hand surgical procedures, both within and beyond the 90-day global billing period. Further investigation and targeted strategies are required to address the observed increase in healthcare utilization. Telehealth implementation is one potential strategy to offset the added resource burden among WC patients in the postoperative period.
